# Tailoring adsorbents for levodopa detection: a DFT study on Pt-encapsulated fullerene systems†

**DOI:** 10.1039/d4ra03526g

**Published:** 2024-08-28

**Authors:** Wendy Maxakato, Miracle N. Ogbogu, Adebayo P. Adeleye, Ismail O. Amodu, Innocent Benjamin, Henry O. Edet

**Affiliations:** a Department of Chemical Sciences, University of Johannesburg Johannesburg South Africa; b Department of Genetics and Biotechnology, University of Calabar Calabar Nigeria; c Department of Chemistry, Federal University of Technology Akure Ondo Nigeria adeleyepauladebayo36@gmail.com; d Department of Chemistry, Marquette University Milwaukee WI USA; e Department of Mathematics, University of Calabar Calabar Nigeria; f Department of Microbiology, University of Calabar Calabar Nigeria benjamininnocent53@gmail.com; g Department of Biochemistry, Cross River University of Technology Calabar Nigeria

## Abstract

Despite its effectiveness in managing the motor symptoms of Parkinson's disease, levodopa therapy is often accompanied by adverse effects that can significantly reduce patients' quality of life. Hence, the need to detect levodopa has escalated among researchers and health experts. Herein, the intricacies of levodopa adsorption were studied using newly tailored fullerene-based adsorbents. All theoretical calculations were performed using the DFT/PBE1PBE/GENECP level of theory. Having modified the surface by Pt-encapsulation followed by functionalization with a functional group (COOH, HCO, NH_2_, NO_2_, and OH), new materials were engineered towards levodopa adsorption. Various theoretical and computational analyses were thoroughly explored to gain insight into the electronic properties, nature of inter- and intra-molecular interactions, strength and phenomenal of adsorption, and the mechanisms of sensing. Adsorption was found to have taken place from the region of the functional groups, where adsorption strength is influenced by the varying electron-withdrawing abilities of the groups. In all cases, the adsorption phenomenon is best described as physisorption. Changes in the dimensions are attributed to the stretching vibration of the bonds on the surface. Also, the small energy gaps within a close range of 0.295 to 0.675 eV exhibited by the materials upon adsorption are an indication of semiconductors. Hence, the functionalized systems hold promise as adsorbents for levodopa molecules, offering valuable insights for future research endeavors.

## Abstract 2

Despite its effectiveness in managing the motor symptoms of Parkinson's disease, levodopa therapy is often accompanied by adverse effects that can significantly reduce patients' quality of life.

## Graphical abstract

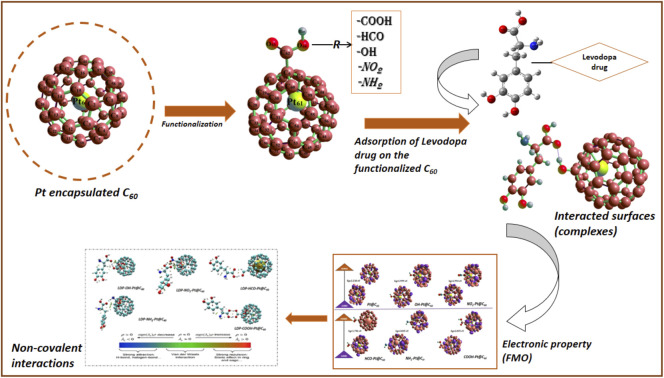

## 1 Introduction

One of the most commonly used drugs in the treatment of Parkinson's disease is levodopa.^1^ This disease, characterized as a neurodegenerative disorder, is accompanied by several motor disabilities (bradykinesia and dyskinesia) and tremors that continuously cause death of nerve cells and impair speech in affected patients.^2,3^ The risks to patients suffering from this disorder can be life threatening, necessitating a critical advancement in pharmacotherapy for the development of various restorative drugs, procedures, and methods for increasing the lifespan of these patients.^4,5^ After approval by the FDA in 1975,^6^ levodopa has been administered for treating Parkinson's disease symptoms due to its potential to bypass various barriers from the blood to the brain.^7^ A major precursor of levodopa is the release of dopamine after the blood–brain barrier has been crossed. The dopamine produced serves as an active stimulant for dopaminergic receptors.^8,9^ Despite its clinical advantages, patients receiving levodopa administration exhibit different side effects, such as hypertensive crises, somnolence, excessive sleepiness, and a reduction in quality of life.^9,10^ In a clinical trial of levodopa in pregnant rabbits, the litters were oddly smaller in size and had developed some skeletal deformities; hence, there is a need for more studies on its effect on humans and possible modifications for more effective results.^11,12^ Due to this effect, the detection of levodopa in the body has become essential to researchers and scientists.

In recent years, the utilization of carbon-based materials has attracted significant attention in various pharmaceutical, industrial, medical, agricultural, and environmental fields due to their unique properties and diverse applications.^13^ Among these materials, C_60_, also known as buckminsterfullerene, has emerged as a promising candidate for surface modification and functionalization due to its exceptional stability, high surface area, and molecular encapsulation capabilities.^14,15^ C_60_ has been utilized in various studies for standard surface modification and functionalization of various substrates to explore its potential as an adsorbent.^16^ The choice of C_60_ as the standard surface in this investigation stems from several advantageous properties such as its excellent chemical stability among many others, which ensures the robustness of the modified surfaces under diverse environmental conditions.^17,18^ Additionally, its high surface area provides ample opportunities for the attachment of functional groups and molecules, facilitating tailored surface modifications.^18^ Moreover, the unique cage-like structure of C_60_ allows for the encapsulation of guest molecules within its framework, offering additional functionalities and enhancing the adsorption properties of the modified surfaces.^19^ Due to this effect, this present work designed new fullerene-based materials by encapsulation and functionalization to form the platinum-encapsulated, X-functionalized fullerene C_60_ adsorbent materials.

According to Martinez, systems that interact strongly with dopamine may be agonistic and may interfere with its delivery at the receptor site because they are good electron donors, such as dopamine.^1^ It was observed that systems with boron form stable structures but might serve better as antipsychotics than delivery systems for dopamine. Additionally, Raghu *et al.* reported that BCN (boron carbonitride) serves as a good electrode material in supercapacitors and electrocatalysts for analysing biological samples for the detection of levodopa.^2^ Duhan and Obrai observed that the fluorescence intensity of nitrogen sulfur codoped graphene quantum dots (NSGQDs) decreased as the concentration of levodopa increased, and this fluorescence showed greater specificity for l-DOPA.^3^ In a study carried out by Alsubaiyel, Amal M., *et al.* on a theoretical investigation of the adsorption and electronic properties of bare and Al-doped C_60_ fullerenes, the adsorption of N_2_O molecule was carried out and the result showed that based on the binding energy of the range of 0.2–41.2 kJ mol^−1^ and the large electronic charge transfer between Al@C_59_ fullerene surface and N_2_O, the Al@C_59_ is a suitable N_2_O gas-sensing material.^20^ In furtherance, Hamid Hadi and coworkers investigated the sensing capabilities of pristine and doped C_60_ fullerene towards methamphetamine (MAT) using density functional theory (DFT). Zn-doped C_60_ fullerene showcased the most favourable sensing attributes as a result of its highest reactivity and adsorption energy of −45.02 kcal mol^−1^. These studies highlighted the potential modified C_60_ fullerene as adsorbents or drug carriers. Hence, they justify the structural modification performed in this present study.^21^

In this comprehensive study, we systematically explored the modification of C_60_ fullerene surfaces through encapsulation and functionalization with various functional groups, including carboxyl (COOH), formyl (HCO), amino (NH_2_), nitro (NO_2_), and hydroxyl (OH) moieties.^19^ The encapsulation carried out involves placing a potential atom inside of the cage-like structure of C_60_ fullerene,^22^ while functionalization, unlike decoration involves bonding functional group to the surface without replacing them, but alters the morphology of the parent surface.^23^ By employing these methods, we aim to tailor the surface properties of C_60_ and investigate their effects towards the detection of levodopa. The adsorption ability of each modified surface was evaluated, aiming to elucidate the influence of different functional groups on the adsorption efficiency and selectivity of the substrates.^24^ Interestingly, a detailed analysis of the adsorption behavior of these functionalized surfaces will provide valuable insights into their potential as an adsorbent for pharmaceuticals.^25,26^ Firstly, analysing the bond lengths and angles provides insights into the structural changes induced by surface modifications, aiding in understanding the underlying mechanisms governing adsorption phenomena.^22^ Levodopa (LDP), an absorbate known for its potential therapeutic applications, served as a test material in this study. Levodopa complexes, including LDP-COOH-Pt@C_60_, LDP-HCO-Pt@C_60_, LDP-NH_2_-Pt@C_60_, LDP-NO_2_-Pt@C_60_, and LDP-OH-Pt@C_60_, were investigated to assess their adsorption ability in adsorption and sensor applications. The novelty of this study lies in its comprehensive investigation of surface modification techniques applied to C_60_ (endohedral encapsulation and functionalization) and their effects on adsorption behaviour. By elucidating the relationship between surface modifications and adsorption properties, this study aims to contribute to the development of advanced materials for various applications, including sensing and catalysis.

## 2 Computational methodology

In the present study, the modified fullerene surface and its complexes were sketched and optimized using Gaussview 09 and Gaussian 16 software at the DFT/PBE1PBE/GENECP level of theory.^27^ It is noteworthy that the optimization achieves the attainment of the potential energy minimum. First, frontier molecular orbital (FMO) analysis was performed, and orbital differences were used to elucidate the conductive and stable properties of the systems.^28^ Additionally, natural bond orbital (NBO) analysis carried out using the NBO 7.1 embedded in the Gaussian package was used to evaluate the stabilization of the systems and charge transfer between the adsorbed levodopa molecule and the modified fullerene systems.^29^ The adsorption of the levodopa molecule on the system was computed using eqn (1).^30^

1 *E*_ads_ = *E*_LDP/Fullerene_ − (*E*_LDP_ + *E*_Fullerene_)

where *E*_LDP/Fullerene_ is the energy of the complex formed between the levodopa molecule and the modified fullerene surface, *E*_LDP_ is the energy of the singly optimized levodopa molecule and *E*_Fullerene_ is the energy of the optimized fullerene surface.^31^ Moreover, the sensor properties, *e.g.*, electrical conductivity, charge transfer and work function, fraction of electron transfer (FET), and back donation, were evaluated to gain insight into the sensing capabilities of the systems.^28^ The topology analysis, which describes the nature of the interaction between the levodopa molecule and the fullerene systems, was obtained from the quantum theory of atom in molecules (QTAIM) and the noncovalent interaction (NCI) carried out in multiwfn, and the visualization was performed using visual molecular dynamics (VMD).^32,33^ Therefore, for constructing an effective adsorbent for levodopa molecules through a theoretical approach, these objectives make it possible to gain insight into the intricacies of adsorption.

## 3 Results and discussion

### 3.1 Structural geometry

The fullerene C_60_ surface, which has a spherical structure and 60 carbon atoms organized in hexagonal and pentagonal rings with high symmetry, was used in this investigation. Afterwards, the C_60_ surface was modified by endohedral encapsulation with a platinum (Pt) atom.^15^ The introduction of the Pt atom was followed by functionalization, with selected functional groups (COOH, HCO, NH_2_, NO_2_, and OH), which induced significant alterations in its geometric properties, particularly concerning bond changes.^19^ The bonding environment changes with the Pt, while the functional groups contribute to the redefined molecular architecture of the fullerene by introducing new bond types, each with its own distinct chemical features.^34^ For example, carboxylate functionalities are imparted by COOH, formyl moieties are introduced by HCO, amino groups are brought by NH_2_, nitro functionalities are added by NO_2_, and hydroxyl groups are brought by OH. These functional groups affect the steric and electrical properties of fullerene in addition to varying the chemical makeup of the bonds.^35^ The combination of these changes yields a complex three-dimensional configuration that reflects the complex interaction between Pt doping and various functional groups. Moreover, the adsorbate levodopa, a precursor of dopamine, is composed of an amino group, a hydroxy group, and a benzene ring.^10^ The levodopa molecule was adsorbed on the functionalized region of the Pt-encapsulated fullerene surface to evaluate the impact of the considered functional groups on the adsorption or sensing of the studied molecule.

**Fig. 1:**
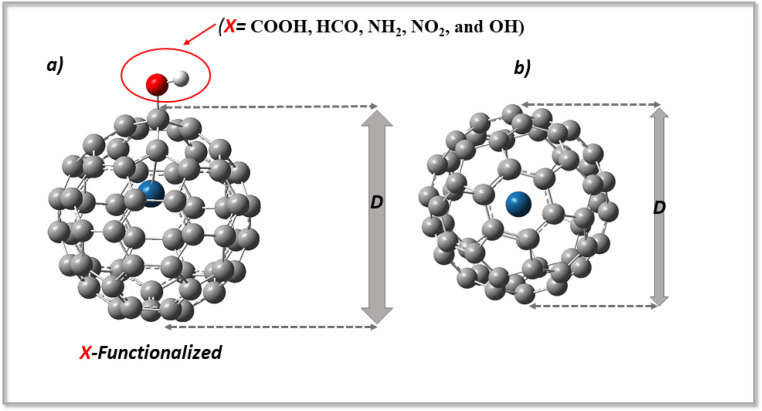
Optimized structures of (a) the adsorbed Cu-Pt@C_60_ and (b) the bare Cu-Pt@C_60_ surface, showing the diameter (*D*) and adsorption distance (*d*).

**Fig. 2:**
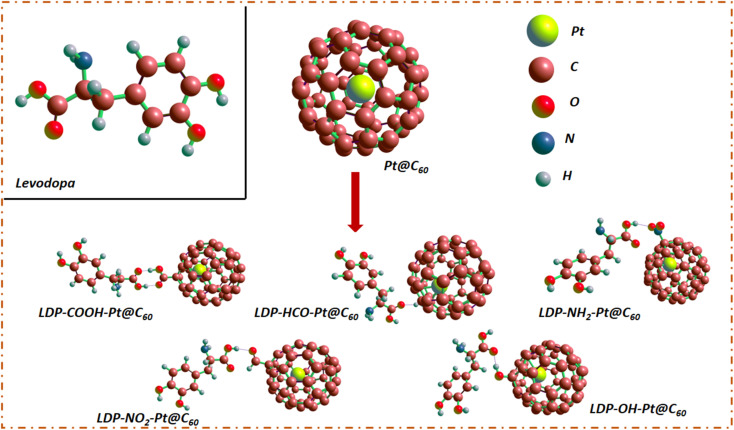
Structural view of the encapsulated fullerene, functionalized models and complexes, and levodopa molecule.

In addition, because the adsorption of levodopa on X-Pt@C_60_ (COOH, HCO, NH_2_, NO_2_, and OH) surfaces may alter the morphology of the surface, the dimensions of the surface may change as a result of functionalization. These changes have been examined to better understand the nature of their vibrations.^18^ Dimensions D were examined before and after functionalization, as well as during adsorption. Changes in the dimensions of the complexes were observed after adsorption, and these changes were found to be significant.^22^ The difference in diameter may be checked so that a change in the surface morphology may be observed. Fig. 1 shows the dimensions of the modified surfaces before and after functionalization, whereas Fig. 2 shows the structures of the systems, levodopa molecules, and complexes formed. Upon functionalization, the dimensions increased from 7.02 Å in Pt@C_60_ to 7.35 Å (4.7%), 7.15 Å (1.9%), 7.39 Å (5.3%), 7.31 Å (4.1%), and 7.38 Å (5.1%), corresponding to COOH-Pt@C_60_, HCO-Pt@C_60_, NH_2_-Pt@C_60_, NO_2_-Pt@C_60_, and OH-Pt@C_60,_ respectively. Upon adsorption, the dimensions increased to 7.35 Å, 7.31 Å, 7.39 Å, 7.31 Å, and 7.39 Å for LDP-COOH-Pt@C_60_, LDP-HCO-Pt@C_60_, LDP-NH_2_-Pt@C_60_, LDP-NO_2_-Pt@C_60_, and LDP-OH-Pt@C_60,_ respectively. These changes in the dimensions are attributed to the stretching vibration of the bonds in which the surfaces are made.

### 3.2 Electronic properties

#### 3.2.1 Quantum descriptors

With the aim of building an effective adsorbent or sensing material for levodopa, a precursor of dopamine, the energies of the frontier molecular orbital, HOMO, and LUMO provide insights into the stable and conductive properties of the proposed fullerene-based adsorbent, Pt@C_60_, with its functionalized counterparts. COOH-Pt@C_60_, HCO-Pt@C_60_, NH_2_-Pt@C_60_, NO_2_-Pt@C_60_ and OH-Pt@C_60_. In line with previous research, the presence of a high energy gap signifies more stable and less reactive systems, whereas a lower energy gap indicates a less stable and more reactive system. In,^28^ frontier molecular orbital analysis was employed to elucidate the properties of their model system for their respective applications.^36,37^ Herein, the studied systems optimized at the DFT/PBE1PBE/GENECP level of theory were evaluated, and the obtained results are presented in Table 1. The pristine Pt@C_60_ surface exhibited an *E*_g_ of 2.238 eV, which indicates its conductive properties. However, upon functionalization with COOH, HCO, NH_2_, NO_2_, and OH groups, a reduction in the energy gap is obtained. HCO-Pt@C_60_ has a much lower energy gap of 1.756 eV, while energy gaps of 1.994 eV and 1.999 eV are observed for NO_2_-Pt@COOH and OH-Pt@COOH, respectively. For the functionalized systems, HCO-Pt@C_60_ was the most conductive, which could be due to the presence of a formyl group, which plays a crucial role in the structure and reactivity of molecules.^38^ Nonetheless, upon the adsorption of levodopa, a more conductive complex is formed, indicating good sensing abilities of the functionalized systems. The LDP-COOH-Pt@C_60_, LDP-HCO-Pt@C_60_, LDP-NH_2_-Pt@C_60_, LDP-NO_2_-Pt@C_60_ and LDP-OH-Pt@C_60_ systems agree with *E*_g_ values of 0.295 eV, 0.306 eV, 0.675 eV, 0.415 eV, and 0.409 eV, respectively. The energy gap (*E*_g_) determines the electrical conductivity of a system in the sense that as the energy gap decreases, the electrical conductivity of the system increases, whereas an increment in the energy gap, causes the electrical conductivity to decrease.^39^ According to the results obtained in tandem with previous literature,^40^ the studied systems exhibit the potential to serve as adsorbents or sensor materials for levodopa.^24,36^ Furthermore, the calculated chemical hardness and softness descriptors are comparable for the systems and complexes, hence supporting the electronic behavior of the systems in this study.

**Table 1:** The calculated HOMO and LUMO energies and quantum descriptors for the isolated and adsorbed systems calculated at DFT/PBE1PBE/GENECP level of theory

System	*E*_HOMO_ eV	*E*_LUMO_ eV	*E*_g_ eV	*μ* (eV)	*η* (eV)	*S* (eV^−1^)	*ω* (eV)
Pt@C_60_	−5.860	−3.622	2.238	4.745	1.119	0.447	10.041
COOH-Pt@C_60_	−9.536	−7.484	2.052	8.510	1.025	0.487	35.295
HCO-Pt@C_60_	−9.456	−7.700	1.756	8.578	0.878	0.569	41.902
NH_2_-Pt@C_60_	−9.469	−7.434	2.035	8.451	1.018	0.491	35.095
NO_2_-Pt@C_60_	−9.753	−7.758	1.994	8.755	0.997	0.501	38.438
OH-Pt@C_60_	−9.544	−7.545	1.999	8.544	0.999	0.500	36.520
LDP-COOH-Pt@C_60_	−7.569	−7.274	0.295	7.421	0.147	3.390	186.705
LDP-HCO-Pt@C_60_	−7.693	−7.387	0.306	7.540	0.153	3.264	185.534
LDP-NH_2_-Pt@C_60_	−7.760	−7.085	0.675	7.423	0.337	1.482	81.644
LDP-NO_2_-Pt@C_60_	−7.799	−7.384	0.415	7.591	0.207	2.410	138.871
LDP-OH-Pt@C_60_	−7.676	−7.267	0.409	7.471	0.205	2.443	136.398

**Fig. 3:**
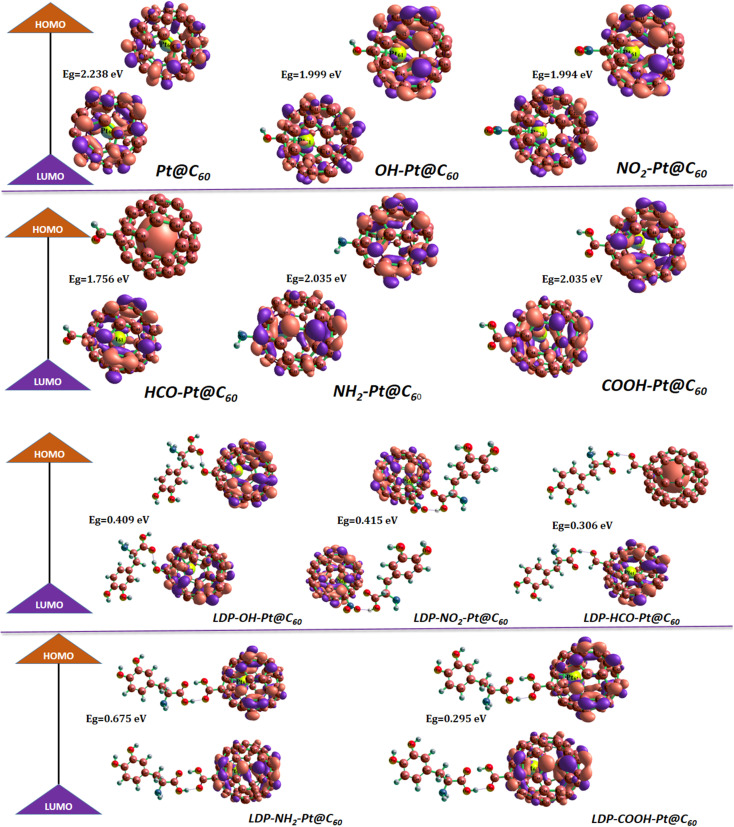
Molecular orbital surface for systems and complexes.

Fig. 3 shows the isosurfaces of the studied systems; these orbitals are evenly distributed across molecular surfaces and complexes, reflecting a balanced electronic configuration. However, an exception is observed in the HCO-Pt-C_60_ complex, where the HOMO is uniquely situated on the encapsulated Pt atom. This typical distribution suggests a pivotal role for the Pt atom in influencing the complex's electronic structure.^22^ Furthermore, when the HCO-Pt-C_60_ surface forms a complex with levodopa, the localization of the HOMO on the Pt atom persists, while the LUMO becomes distributed around the surface. This implies two major things (electron donor and orbital overlap). Pt atom having a localized HOMO acts as an electron donor to levodopa. This creates forces of attraction between the positively charged Pt and functional groups in levodopa that can accept electrons, thereby encouraging adsorption. Also, the spread-out LUMO of the surface result in orbital overlap in levodopa, leading to the formation of new bonding interactions that hold the levodopa molecule to the surface.

#### 3.2.2 NBO analysis

Conversely, natural bond orbital (NBO) analysis was carried out to confirm the type of donor–acceptor orbital interaction that exists between the adsorbed levodopa molecules on the tailored surfaces.^39^ The NBO method is a strategy for simplifying the difficult Schrödinger equation into an understandable chemical bonding idea, and the intermolecular charge transfer and electron delocalization that occur between the adsorbent and adsorbate are also well highlighted by NBO analysis.^41^ However, the degree of interaction between the donor and acceptor orbitals is quantified by the stabilization energy *E*^2^. A greater intensity of interaction between these orbitals corresponds to a greater stabilization energy.^36,39^ In accordance with prior computational research, the stabilization energy for the most contributing donor–acceptor orbitals in the studied systems, and the results obtained are presented in Table 2. According to the results, the π and LP transitions are dominant in the studied systems. LDP-OH-Pt@C_60_ has a higher *E*^2^ of 2509 kcal mol^−1^ with a transition between LP C1 and LP* C58, which is a charge delocalization resulting from charge transfer from lone pair of carbon atom to anti-bonding character of lone pair of carbon atom, whereas the lowest *E*^2^ of 11.66 kcal mol^−1^ is observed for Pt@C60 between π C59–C60, π* C54 and C55. For the functionalized systems, a comparable stabilization energy is observed, except for COOH-Pt@C_60_, which has the highest *E*^2^ herein, confirming the findings from the FMO analysis. The decreasing order of stabilization energies for the fullerene-based systems and complexes is as follows: LDP-OH-Pt@C_60_ > LDP-COOH-Pt@C_60_ > LDP-NH_2_-Pt@C_60_ > COOH-Pt@C_60_ > LDP-NO_2_-Pt@C_60_ > LDP-HCO-Pt@C_60_ > NO_2_-Pt@C_60_ > OH-Pt@C_60_ > HCO-Pt@C_60_ > Pt@C_60_. In this context, it is noteworthy that the systems and complexes under consideration exhibit good attributes of a sensing material, wherein the LDP-OH-Pt@C_60_ complex will be easily stabilized during adsorption as compared to its studied counterparts.

**Table 2:** Most contributing second-order perturbation energies for the studied systems calculated at DFT/PBE1PBE/GENECP level of theory

Systems	Donor (*i*)	Acceptor (*j*)	*E* ^2^ kcal mol^−1^	*E*(*i*)–*E*(*j*)	*F*(*i*,*j*)
Pt@C_60_	π C_59_–C_60_	π* C_54_–C_55_	11.66	0.32	0.055
COOH-Pt@C_60_	LP O_64_	LP* H_65_	519.43	0.68	0.531
HCO-Pt@C_60_	π C_4_–C_9_	π* C_10_–C_11_	232.10	0.01	0.076
NH_2_-Pt@C_60_	π* C_1_–C_58_	π* C_16_–C_57_	238.68	0.01	0.077
NO_2_-Pt@C_60_	π* C_54_–C_55_	π* C_45_–C_53_	244.00	0.01	0.076
OH-Pt@C_60_	π* C_1_–C_58_	π* C_16_–C_57_	243.55	0.01	0.077
LDP-COOH-Pt@C_60_	LP C_1_	LP* C_58_	2013.87	0.02	0.161
LDP-HCO-Pt@C_60_	LP O_85_	LP* H_86_	513.55	0.65	0.519
LDP-NH_2_-Pt@C_60_	LP C_4_	LP* C_9_	2156.44	0.02	0.163
LDP-NO_2_-Pt@C_60_	LP O_85_	LP* H_86_	515.35	0.65	0.526
LDP-OH-Pt@C_60_	LP C_1_	LP* C_58_	2509.66	0.01	0.161

### 3.3 Adsorption studies

The adsorption of levodopa (LDP) on X-functionalized Pt-encapsulated fullerenes (X-Pt@C_60_, where X = COOH, HCO, NH_2_, NO_2_, and OH) was investigated in this study. The optimized structures for LDP adsorption on the functionalized surfaces are visualized in Fig. 2, which shows the structural geometry. The calculated adsorption energies show physisorption phenomena of adsorption,^42^ which is due to the positive adsorption energy obtained, calculated within a close range of 326.3 to 340.7 kcal mol^−1^. The results obtained are summarized in Table 3. The presence of weak interactions between the adsorbate and adsorbent was identified from the magnitude of the adsorption energy. The least positive adsorption energy of 326.3 kcal mol^−1^ attributed to LDP-COOH-Pt@C_60_ shows relatively better detection of levodopa than their counterparts. This is because the farther the adsorption energy is from the negative, the weaker it becomes.^31^ In the present work, LDP-NH_2_-Pt@C_60_ showed the largest positive adsorption energy of 340.7 kcal mol^−1^, suggesting that it had the weakest detection energy for levodopa. Since the energies are relatively close, the detection strength for levodopa follows the pattern LDP-COOH-Pt@C_60_ < LDP-OH-Pt@C_60_ < LDP-NO_2_-Pt@C_60_ < LDP-NO_2_-Pt@C_60_ < LDP-NH_2_-Pt@C_60_, which is a decreasing order of the detection strength (see Table 3). Furthermore, structural modification by functionalization with the –COOH functional group resulted in better detection, followed by functionalization with the –OH functional group and then functionalization with the –NO_2_ functional group, as shown in the aforementioned pattern. In general, the presence of close adsorption range of 326.3 to 340.7 kcal mol^−1^ indicates that all tailored materials exhibit a similar pattern of adsorption strength.

**Table 3:** The summarized results of the computed adsorption energy calculated *via* the DFT/PBE1PBE/GENECP level of theory

Complexes	*E*_complex_	*E*_surface_	*E*_gas_	*E*_ad_ (Ha)	*E*_ad_ (eV)	*E*_ad_ (kcal mol^−1^)
LDP-COOH-Pt@C_60_	−3293.664154	−2589.674077	−704.510086	0.5200	14.15	326.3
LDP-HCO-Pt@C_60_	−3218.511165	−2514.540275	−704.510086	0.53922	14.67	338.4
LDP-NH_2_-Pt@C_60_	−3160.657245	−2456.690070	−704.510086	0.5429	14.77	340.7
LDP-NO_2_-Pt@C_60_	−3309.537076	−2605.565038	−704.510086	0.5381	14.64	337.6
LDP-OH-Pt@C_60_	−3180.497622	−2476.520130	−704.510086	0.5326	14.49	334.2

### 3.4 Visual studies

#### 3.4.1 QTAIM

The quantum theory of atoms-in-molecules (QTAIM) proposed by Bader has become an important analysis technique for the study of the intra- and intermolecular interactions occurring in atoms of a compound.^35^ This analysis is also regarded as an analysis of weak interactions and consists of different variables that describe the system. Its variables range from the bond critical points (BCPs), which indicate the bonds present, and improve the calculation and prediction of other parameters of the QTAIM. Other topological parameters include the electron density (*H*(*r*)), Laplacian of electron of electron density ∇^2^*ρ*(*r*), Eigenvalues of Hessian, and ellipticity of electro density (*e*), among others. Table 4 encompasses the parameters of the QTAIM analysis as observed in this study. The highest *ρ*(*r*) values observed in the complexes are 0.799 a,u at H_73_–N_88_ for LDP-COOH-Pt@C_60_, 0.308 a.u. at H_86_–O_63_ for LDP-HCO-Pt@C_60_, 0.577 a.u. at C_9_–H_81_ for LDP-NH_2_-Pt@C_60_, 0.827 a.u. at O_84_–C_9_ for LDP-NO_2_-Pt@C_60_ and 0.433 a.u. at H_63_–O_83_ for LDP-OH-Pt@C_60_. As stated in the literature, *ρ*(*r*) > 0; ∇^2^*ρ*(*r*) > 0 indicates the presence of a potent noncovalent bond, *ρ*(*r*) < 0; ∇^2^*ρ*(*r*) < 0 indicates the presence of a covalent interaction, and when *ρ*(*r*) > 0; ∇^2^*ρ*(*r*) < 0 indicates an existing partial covalent interaction between and within the studied complexes.^43^

**Table 4:** Results for selected QTAIM parameters of the studied complexes calculated at DFT/PBE1PBE/GENECP level of theory^a^

System	Bond	BCP	*ρ*(*r*)	∇^2^*ρ*(*r*)	*G*(*r*)	*K*(*r*)	*V*(*r*)	*H*(*r*)
LDP-COOH-Pt@C_60_	C_62_–C_6_	152	0.232	−0.468	0.578	0.175	−0.233	−0.175
	H_73_–N_88_	96	0.799	0.354	0.662	−0.223	−0.439	0.223
	C_80_–C_84_	126	0.240	−0.506	0.608	0.187	−0.248	−0.187
LDP-HCO-Pt@C_60_	H_72_–N_87_	93	0.128	0.542	0.109	−0.265	−0.825	0.265
	O_84_–H_64_	148	0.167	0.762	0.159	−0.318	−0.127	0.318
	H_86_–O_63_	94	0.308	0.109	0.264	−0.786	−0.256	0.786
LDP-NH_2_-Pt@C_60_	C_9_–O_84_	221	0.406	0.166	0.306	−0.109	−0.197	0.109
	H_64_–O_84_	247	0.195	0.886	0.193	−0.283	−0.165	0.283
	C_9_–H_81_	195	0.577	0.231	0.415	−0.162	−0.253	0.162
LDP-NO_2_-Pt@C_60_	O_84_–N_62_	220	0.100	0.464	0.905	−0.255	−0.650	0.255
	H_86_–O_64_	248	0.252	0.925	0.219	−0.122	−0.207	0.122
	O_84_–C_9_	180	0.827	0.410	0.763	−0.262	−0.501	0.262
LDP-OH-Pt@C_60_	C_9_–O_83_	238	0.289	−0.744	0.986	0.285	−0.383	−0.285
	H_63_–O_83_	216	0.433	0.154	0.395	0.958	−0.405	−0.958
	C_78_–N_86_	241	0.263	−0.512	0.130	0.258	−0.387	−0.258

a The units of the parameters in Table 4 are as follows: *P*(*r*), *e*/Ang^3^; ∇^2^*ρ*(*r*), *e*/Ang^3^; *G*(*r*) and *V*(*r*) have units of eV; *H*(*r*) has a unit of eV Ang^3^/*e*.

Here, as shown in Table 4, all *ρ*(*r*) values for the systems are greater than zero and positively charged, and the Laplacian of the electron density withholds a larger number of positive values greater than zero. This indicates a stronger presence of noncovalent interactions within the studied complexes, with LDP-NO_2_-Pt@C_60_ withholding the highest value and LDP-COOH-Pt@C_60_ having a minimal interaction. The positive values of the Laplacian of electron density show that the complexes exhibit an electrostatic nature of interaction.^44^ The degree of structural stability within the complexes can be estimated by the ellipticity of the electron density.^1,3^ The greater the ellipticity of the electron density is, the lower the interaction stability. On average, the values are 0.45 a.u. for LDP-COOH-Pt@C_60_, 0.008 a.u. for LDP-HCO-Pt@C60, 0.567 a.u. for LDP-NH_2_-Pt@C_60_, 0.785 a.u. for LDP-NO_2_-Pt@C_60_ and 0.112 a.u. for LDP-OH-Pt@C_60_. Furthermore, the eigenvalues of the Hessian matrix (*λ*_1_/*λ*_3_) are less than one, which implies the presence of stronger intermolecular forces on the interacting surfaces. The electron density of all the complexes except LDP-NH_2_-Pt@C_60_ is greater than 0.5 a.u., which, according to previous literature, indicates that the systems exhibit delocalized electrons within their molecules.

#### 3.4.2 Reduced density gradient (RDG) analysis

**Fig. 4:**
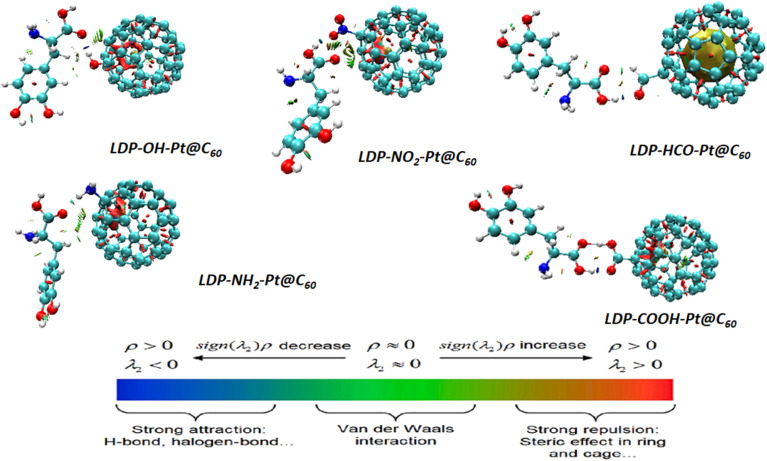
3D-NCI plots for the studied complexes.

Aside from investigating the electron charge transfer within molecules, it is paramount to investigate the nature of the bond in the molecules of a system. The forces of attraction can be categorized as weak or strong and exist in a variety of forms, where the van der Waals force is a weak force and the steric force is a strong force of attraction.^45^ Reduced density gradient analysis is referred to as a noncovalent study (NCI) because of its specificity in identifying weak interactions required in an adsorption study to understand the sensation and adsorption level of the levodopa molecule using various modified systems.^46^ The application of this technique has spread across various fields and research interests, such as environmental and toxicology studies, power industries, and drug delivery.^46,47^ By employing a computational approach, NCI visualization was carried out, as shown in Fig. 4. The VMD application was utilized, and its isosurfaces appeared in different colour ranges, each having valuable information that aided the clarity of this study.

These loop-colored isosurfaces enhance the comprehension and graphical representation of the interactions occurring within and between systems, as reported in the QTAIM study. By analyzing the 3D RDG plot, valuable insights into the nature and strength of the forces governing adsorption can be gained. Herein, red-colored regions represent a steric force of attraction known as an intense kind of attraction. The green region indicates the degree of van der Waals dispersion forces occurring in the molecules, and the blue isosurface patterns indicate the hydrogen bonds. All these forces contribute to the determination of the degree of adsorption between the adsorbent and adsorbate. In Fig. 4, complexes LDP-OH-Pt@C_60_ and LDP-NH_2_-Pt@C_60_ appear to have a green colour that is sparsely scattered around the region of the levodopa compound, and this observation represents the presence of van der Waals dispersion. Furthermore, the LDP-NO_2_-Pt@C_60_ complex is characterized by van der Waals interactions with a slight steric force owing to the occurrence of greener isosurface and a slight appearance of red loops. Analysis of the red zones in the NCI plot is crucial for pinpointing the locations of high-strength attractive interactions responsible for anchoring the adsorbate molecule onto the surface. For the LDP-HCO-Pt@C_60_ complex, the levodopa compound is marginally surrounded by red and blue colours, which may be caused by the electronegativity of the oxygen atom in the HCO functional group, whereas the platinum metal is primarily surrounded by a green colour. The –COOH-functionalized surface exhibited a sparse distribution of red and blue colours within and between upon interaction with our studied pharmaceutical pollutant. According to the observations in this study, the LDP-OH-Pt@C_60_, LDP-NO_2_-Pt@C_60_, LDP-NH_2_-Pt@C_60_, and LDP-COOH-Pt@C_60_ complexes are the most suitable systems for the adsorption of levodopa. In general, the presence of red regions near the interface between the levodopa and the tailored fullerene surfaces indicates strong attractive interactions that contribute significantly to adsorption strength. The presence of green regions, suggests additional dispersion forces contributing to the overall attraction brought by the adsorption configurations. Finally, specific hydrogen bonds further stabilizing the adsorbed levodopa can be pinpointed *via* the blue region.

### 3.5 Sensor properties

Categorizing sensor mechanisms based on their ability to provide insights into which materials are suitable for use as detectors and sensors in various systems is a step toward material commercialization. Oftentimes, it can be bothersome to show that some materials show a common tendency as sensors; however, their applicability has become a concern in engineering.^43^ In the present section, an in-depth examination of the applicability of the labelled systems is carried out. The mechanisms discussed include electrical conductivity, charge transfer mechanism, work function, FET, and back donation.

#### 3.5.1 Electrical conductivity

Electrical conductivity is the transfer of current from one end to the other over a surface in an electric field.^48^ The formula employed for the electrical conductivity *σ*, holds at temperature *T*, energy gap *E*_g_, constant *K*, and finally Boltzmann constant *B*, as presented in eqn (2):^49,50^

2 *σ* = *AT*^2/3^*e*^(*E*_g_/2*KT*)^

The conductivity can be related to the energy gap, so a small energy gap results in a high conductivity.^37^Table 5 shows how the energy gap changes as well as the percentage change in the energy gap. Due to the large percentage change in the energy gap obtained, it can be confirmed that the greatest change observed is due to the large changes as a result of the adsorption process. This in turn indicates the enhanced conductivity of the studied systems.

**Table 5:** Sensor properties calculated *via* the DFT/PBE1PBE/GENECP level of theory^a^

Systems	*E*_ads_	*ϕ*	Δ*E*_g_	% Δ*E*_g_	*Q*_t_	Δ*E*_back-donation_	Δ*N*
LDP-COOH-Pt@C_60_	326.3	7.421	−0.8562	−85.62	−1.225	−0.03675	−0.47856
LDP-HCO-Pt@C_60_	338.4	7.540	−0.8257	−82.57	−1.033	−0.03825	−0.37628
LDP-NH_2_-Pt@C_60_	340.7	7.423	−0.6683	−66.83	1.097	−0.08425	−0.35003
LDP-NO_2_-Pt@C_60_	337.6	7.591	−0.7919	−79.19	−0.908	−0.05175	−0.45978
LDP-OH-Pt@C_60_	334.2	7.471	−0.7954	−79.54	1.208	−0.05125	−0.42598

a The units of *E*_ad_, Δ*E*_g_, *ϕ*, and Δ*E*_back-donation_ are in electron volt (eV). The *Q*_t_ has a unit of electron (e), and finally the % Δ*E*_g_ has its unit in percentage. Δ*N* is dimensionless.

#### 3.5.2 Charge transfer analysis and work function

**Fig. 5:**
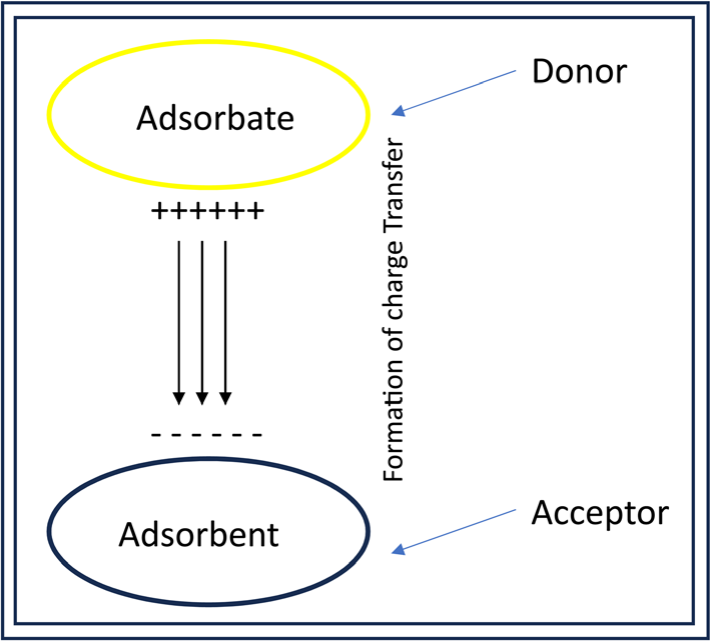
Schematic presentation of the formation of charge transfer between adsorbate and adsorbent.

Qt is a result of inquiring into the distribution of electron densities. Herein, the natural charges of the adsorbent before and after adsorption were used to investigate the charge transfer mechanism. Eqn (3) (ref. 51) was used in the computation of the charge transfer (*Q*_t_), and the results are summarized in Table 5. The schematic presentation of the formation of charge transfer between adsorbate and adsorbent is visualized in Fig. 5.

3 *Q*_t_ = *Q*_adsorption_ − *Q*_isolated_

Understanding this mechanism can be traced from the adsorbent to the adsorbate, and the other way around might be done using the plus and minus signs accompanying the magnitude of the charge transfer. Generally, electron transfer from the adsorbate to the adsorbent can be read as a negative *Q*_t_, whereas a positive *Q*_t_ translates to the transfer of electrons from the adsorbent to the adsorbate.^52^ In this work, electrons were transferred from the levodopa molecule to the adsorbents, except for LDP-NH_2_-Pt@C_60,_ where electrons were transferred the other way around. Furthermore, it can be confirmed from Table 5 that the *ϕ* values computed are within a relatively small range of 7.421–7.591 eV, indicating little to no significant difference among the complexes. These results further show that the sensing attributes associated with the work function are nearly uniform among the labelled systems.

#### 3.5.3 Fraction of electron transfer (FET) and back donation

The region of electron transfer from the functionalized surface to the levodopa gas can be understood using the fraction of electron transfer (FET) mechanism,^23,52^ which can be written as Δ*N*. Gómez and coworkers established the possible contact that can exist between the adsorbent and the levodopa molecule during the adsorption process, thereby being controlled by the use of the electrical back-donation mechanism.^53^ Notably, the backward movement of electrons from levodopa to the labelled functionalized surface is known as back donation, and it is denoted as Δ*E*. The results computed at the DFT/PBE1PBE/GENECP level of theory are summarized in Table 5. Based on Pearson's theory, eqn (4) and (5) show how quantum descriptors can be related to Δ*E* and Δ*N*, respectively.^54,55^4
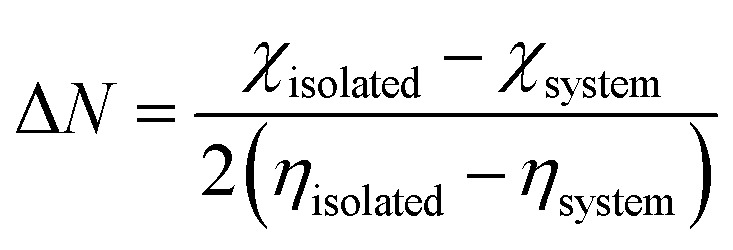
5
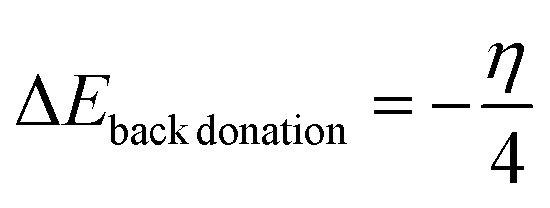


According to eqn (4), a high Δ*N* may be achieved when the chemical hardness is low, which can further increase electron transfer during the adsorption process. Owing to a decrease in the tendency for charge transfer, the barrier to electron transfer can be improved. As expected for a material suitable for use as a sensor or detector device, back-donation is negative (that is, Δ*E* < 0), and the chemical hardness is positive (*η* > 0).^44^Table 5 shows that the Δ*E* values are less than zero, indicating that the studied materials are good sensor materials. It is computationally necessary to say that a decrease in the *η* value will in turn reflect a corresponding decrease in stability. Additionally, high electron transfer from the surface to the adsorbate implies strong adsorption of the levodopa molecule, which can in turn increase stability, provided that there is an increase in back donation.^42,44^ LDP-COOH-Pt@C_60_, LDP-NO_2_-Pt@C_60_, and LDP-OH-Pt@C_60_ exhibited relatively high FET values of −0.47856, −0.45978, and −0.42598, respectively. This result confirmed the stability and relatively better adsorption in the aforementioned systems, confirming that the surfaces are suitable materials for detecting levodopa molecules, especially the COOH-Pt@C_60_ surface.

### 3.6 Density of state (DOS) analysis

In computational studies, within the density functional theory framework, the density of states (DOS) analysis is vital in investigating and understanding the charge behaviors, flow, and transfer within atoms in a compound or material. The charge studies provide insights into the electron distribution which accounts for a percentage degree of interactions between the adsorbate and the adsorbent. Here, the energy sum in the materials is deciphered and the electrons transition from a ground state to an excited state is utilized in depicting the density of states level which directly affects and accounts for a material's behavior. Furthermore, the DOS analysis in a sense possesses a positive correlation with the patterns of electron shift in the HOMO–LUMO plots.

**Fig. 6:**
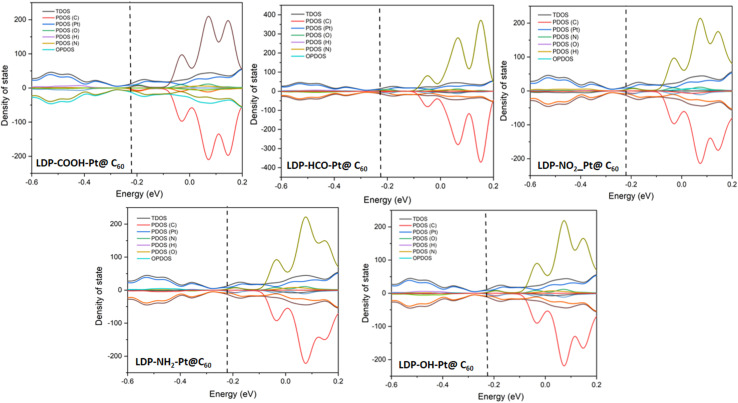
Density of state plots showing the position of the TDOS, PDOS, and OPDOS of the studied surfaces upon adsorption.

The DOS plots for all complexes are presented in Fig. 6. All surfaces possess a good percentage of carbon atoms and a platinum atom arising from the doping effect. The surfaces other than Pt-@C_60_ possessed a range of atoms such as O, H, and N. In the plots. The atoms are represented in a colour tag with the total density of states (TDOS) above and the overlapping partial density of states (OPDOS) below, bearing the partial density of states (PDOS) between. The hydrogen atom possessed the least contribution and carbon the highest contribution with C falling within the lower range as compared to the TDOS. In the interaction plots, O possessed the peak in LDP-HCO-Pt@C_60_, LDP-NO_2_-Pt@C_60_, LDP-NH_2_-Pt@C_60_, LDP-OH-Pt@C_60_, interactions, and H possessed the highest in LDP-COOH-Pt@C_60_. For all complexes, the Fermi energy level was present which is denoted as the average of the sum of the highest occupied molecular orbital (HOMO) and the lowest occupied molecular orbital (LUMO). Existing literature established that the distance of the Fermi energy level to the energy gaps affects the mobility of electrons.^56^ A closer distance positions a faster transfer of electrons and *vice versa*. For all complexes plots, the Fermi-energy falls within the range of −0.21 to −0.23 e.V with an energy gap range of 0.2 to 0.7 eV for interactions and 1.7–2.3 eV for surfaces. A closer distance in the Fermi-energy and energy gap is observed in the interacted surfaces which denotes a better electron activity likely occurred. Therefore, the results show an average distribution of charges has positively influenced the adsorption rate of the levodopa molecule on the adsorbents.

### 3.7 Molecular electrostatic potential (MESP)

**Fig. 7:**
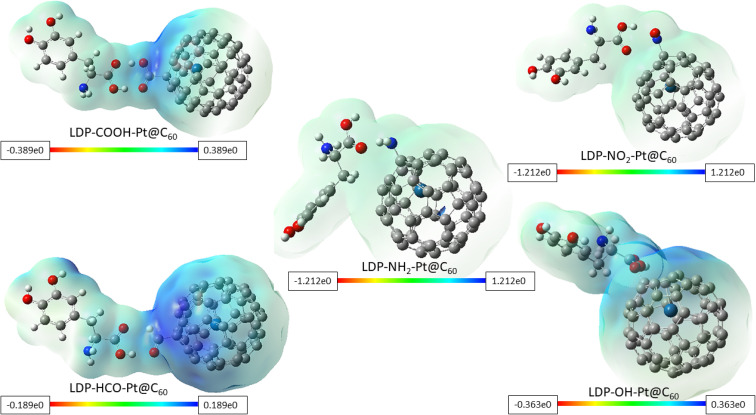
Molecular electrostatic potential (MESP) maps of the studied surfaces upon adsorption.

As a valuable tool in molecular modelling of adsorption, MESP helps in understanding the distribution of electron density around a molecular system. This distribution influences how the molecule interacts with other molecules, including how strongly adsorption is onto a surface. Due to a negatively charged region of one molecule approaching a positively charged region of another, an attractive force arises as a result of opposite charges.^57^ This analysis enables one to identify these complementary regions and predict potential sites for favorable electrostatic interactions during adsorption. It also aids in predicting potential locations for hydrogen bonding. The hydrogen bonds are formed when a hydrogen atom bonded to a highly electronegative atom (like N, O, or F) interacts with another highly electronegative atom in a nearby molecule. Areas of high electron density are revealed around these electronegative atoms, thus showcasing potential sites for hydrogen bond formation during adsorption. Understanding the dipole–dipole interactions that occur between molecules with permanent electric dipoles contributes to the adsorption strength. Fig. 7 visualized the MESP maps for the investigated surfaces upon adsorption. The color scheme of the MESP maps is as follows: red color signifies electron-rich, partially negative charge; blue denotes electron-deficient, partially positive charge; yellow corresponds with slightly rich in electrons; and green implies neutral.^58^ Electropositive and electronegative regions are encoded in blue and red colors respectively.^59^ In Fig. 7, around complexes LDP-COOH-Pt@C_60_, LDP-HCO-Pt@C_60_, and LDP-OH-Pt@C_60_, blue color can be seen near the area where –COOH, –HCO, and –OH groups functionalization took place, showing electrophilic characteristic. This is due to a result of the oxygen atom present in the functional groups, thereby creating a region of attractive electrostatic interaction. In addition, it can be inferred that electron density is in the aforementioned functional groups. The faint blue-green color observed in LDP-NH_2_-Pt@C_60_, and LDP-NO_2_-Pt@C_60_, around a region between the green and blue colors as shown in the maps (see Fig. 7), indicates a slightly deficient in electrons, approaching neutral. This suggests a combination of weak attractive and repulsive forces between the levodopa and the NH_2_-Pt@C_60_ and NO_2_-Pt@C_60_ adsorbents. This also reveals the presence of weak dispersion forces between the two interacting parties, arising from temporary fluctuations in electron density. Owing to the obtained results, it can be deduced in general, that adsorption of levodopa to the tailored surfaces is carried out from the region of the functional groups and adsorption strength is influenced by the varying electron-withdrawing abilities of the groups.

## 4 Conclusions

Investigation of the endohedral doping of a Pt atom on fullerene (C_60_) followed by functionalization with specific groups (COOH, HCO, NH_2_, NO_2_, and OH) as adsorbents for levodopa was carried out using DFT/PBE1PBE/GENECP level of theory. Various theoretical analyses were thoroughly explored to gain insight into the electronic properties, nature of inter- and intra-molecular interactions, strength and phenomenal of adsorption, and the mechanisms of sensing. The following deductions have been drawn:

(I) The dimension elongated upon adsorption to 7.35 Å, 7.31 Å, 7.39 Å, 7.31 Å, and 7.39 Å for LDP-COOH-Pt@C_60_, LDP-HCO-Pt@C_60_, LDP-NH_2_-Pt@C_60_, LDP-NO_2_-Pt@C_60_, and LDP-OH-Pt@C_60,_ respectively. These changes in the dimensions are attributed to the stretching vibration of the bonds in which the surfaces are made.

(II) All formed complexes exhibit of small energy gaps of 0.295 eV, 0.306 eV, 0.675 eV, 0.415 eV, and 0.409 eV, corresponding to the LDP-COOH-Pt@C_60_, LDP-HCO-Pt@C_60_, LDP-NH_2_-Pt@C_60_, LDP-NO_2_-Pt@C_60_, and LDP-OH-Pt@C_60_ complexes. This result indicates that electrons behave similarly in these materials, and exhibit a characteristic property of semi-conductors.

(III) The decreasing order of stabilization energies follows: LDP-OH-Pt@C_60_ > LDP-COOH-Pt@C_60_ > LDP-NH_2_-Pt@C_60_ > COOH-Pt@C_60_ > LDP-NO_2_-Pt@C_60_ > LDP-HCO-Pt@C_60_ > NO_2_-Pt@C_60_ > OH-Pt@C_60_ > HCO-Pt@C_60_ > Pt@C_60_. As compared to its studied counterparts, the LDP-OH-Pt@C_60_ will be easily stabilized during adsorption.

(IV) Since the energies are relatively close, the detection strength for levodopa follows the pattern LDP-COOH-Pt@C_60_ < LDP-OH-Pt@C_60_ < LDP-NO_2_-Pt@C_60_ < LDP-NO_2_-Pt@C_60_ < LDP-NH_2_-Pt@C_60_, which is a decreasing order of the detection strength.

(V) During AIM evaluation, in most cases, *ρ*(*r*) > 0 and ∇^2^*ρ*(*r*) > 0 are obtained, indicating a stronger presence of noncovalent interactions which is in turn suitable for adsorption.

(VI) Deduction from MESP maps shows that adsorption of levodopa to the tailored surfaces is carried out from the region of the functional groups and adsorption strength is influenced by the varying electron-withdrawing abilities of the groups. This result was further substantiated using the 3D RDG maps in the NCI analysis.

(VII) Transfer of electrons occurred from the levodopa molecule to the adsorbents, except for LDP-NH_2_-Pt@C_60,_ where electrons were transferred the other way around. LDP-COOH-Pt@C_60_, LDP-NO_2_-Pt@C_60_, and LDP-OH-Pt@C_60_ exhibited relatively high FET values of −0.47856, −0.45978, and −0.42598, respectively, showcasing the stability and propensity of the labelled surfaces towards levodopa adsorption.

## Data availability

All data are contained within the manuscript and manuscript ESI.† The available software used for the computational studies includes: Gaussian16 and GuassView6.0.16. Other software codes include: Chemcraft, Multiwfn analyser, and the Visual molecular dynamic (VMD) program, available at http://sobereva.com/multiwfn/ and https://www.ks.uiuc.edu/Research/vmd/, respectively.

## Conflicts of interest

No conflicts of interest.

## Supplementary Material

RA-014-D4RA03526G-s001
