# Supplementary material for: Tailoring adsorbents for levodopa detection: a DFT study on Pt-encapsulated fullerene systems
Source: RSC Adv. 2024 Aug 28;14(37):27424–37. doi: 10.1039/d4ra03526g (PMC11353775; doi:10.1039/d4ra03526g)
Supplement: RA-014-D4RA03526G-s001 [file RA-014-D4RA03526G-s001.pdf]

Supplementary Information (SI) for RSC Advances.
This journal is © The Royal Society of Chemistry 2024

Supporting Information

Wendy Maxakato[a], Innocent Benjamin, Miracle N. Ogbogu[c], Henry O. Edet[b], Ismail O. Amodu[d], and Adedapo S. Adeyinka[a]

Table S1. Results for some QTAIM parameters; Ellipticity bond, Electron localization function (ELF) and Eigenvalues

ε	ELF	λ1	λ2	λ3	λ1/ λ3
0.016	0.950	0.395	-0.435	-0.428	-0.923
1.290	0.188	-0.448	0.411	-0.469	0.955
0.0544	0.950	0.378	-0.454	-0.430	-0.879
0.168	0.326	0.805	-0.121	-0.142	-5.669
0.052	0.762	0.117	-0.197	-0.208	-0.563
0.024	0.970	0.212	-0.522	-0.509	-0.417
1.003	0.930	-0.108	0.198	-0.217	0.498
0.019	0.421	0.143	-0.272	-0.277	-0.516
0.679	0.162	0.307	-0.477	-0.284	-1.081
0.897	0.214	0.593	-0.844	-0.445	-1.333
0.052	0.743	0.169	-0.374	-0.394	-0.429
1.406	0.159	0.495	-0.601	-0.250	-1.980
0.157	0.931	0.279	-0.475	-0.549	-0.509
0.017	0.130	-0.810	0.315	-0.797	1.016
0.050	0.850	0.410	-0.450	-0.472	-0.869
